# Editorial: Metabolic disorders as risk factors for osteoarthritis and targeted therapies for this pathology

**DOI:** 10.3389/fendo.2024.1540942

**Published:** 2025-01-06

**Authors:** Giuseppe Lisco, Hee Geun Jo, Graziana Colaianni

**Affiliations:** ^1^ Department of Interdisciplinary Medicine, School of Medicine, University of Bari Aldo Moro, Bari, Italy; ^2^ Department of Herbal Pharmacology, College of Korean Medicine, Gachon University, Seongnam-si, Republic of Korea; ^3^ Department of Precision and Regenerative Medicine and Ionian Area (DiMePRe-J), University of Bari Aldo Moro, Bari, Italy

**Keywords:** osteoarthritis, diabetes mellitus, obesity, metabolic syndrome, mesenchymal stem cells, editorial

Osteoarthritis (OA) is a chronic polyarticular degenerative disease characterized by progressive joint destruction and loss of function (1). Primitive osteoarthritis (POA) affects around 3.5% of the general population (2) and represents the most common cause of disability in older people (3). It results from a combination of risk factors, mechanical overload, and altered joint mechanics (4). Common risk factors of POA include aging, weight excess, muscle weakness, a sedentary lifestyle, and recurring or persisting joint injuries (5).

POA is characterized by a marked rearrangement of the articular cartilage, resulting from focal erosion extending from the articular cartilage surface to the subchondral bone, chondrocyte hypertrophy, and apoptosis, cartilage overgrowth outside the articular surface with formation of osteophytes, and ossification of subchondral bone. These alterations are mirrored by detrimental macroscopic changes, such as cartilage and ligament degeneration, secondary synovitis, and overall joint rearrangement, prompting relevant deformities (6). Given that the articular cartilage tissue has a scarce regenerative property (7), articular damage in POA results in an irreversible derangement ranging from a minimal and stable disease to a severe and progressively deforming OA (8).

Epidemiological evidence indicates POA is frequent, and its clinical presentation is anticipated in patients with obesity, diabetes mellitus, and metabolic syndrome (9). Besides weight excess, one of the most relevant but not the only risk factors associated with POA, recent investigation has highlighted other influential mechanisms associated with POA in patients with metabolic diseases, including hyperglycemia and related metabolic pathways, insulin resistance, chronic low-grade inflammation from the adipose tissue, oxidative stress, and altered lipid metabolism (10, 11). Therefore, treatments aiming to control glucose levels, improve insulin sensitivity, reduce body weight, ameliorate body composition, and reduce adipose tissue and systemic inflammation (12–14) may have a therapeutic rationale as preliminary studies suggest (15, 16).

The Research Topic focused on the role of metabolic disorders in the pathogenesis of POA, aiming to improve the knowledge of the pathophysiology, diagnostic biomarkers, and novel treatments in OA.

A comprehensive mechanistic review by Adam et al. described the putative mechanisms illustrating the relationship between glucose and lipid dysmetabolism and the activity of chondrocytes. First, lipid and glucose accumulation in chondrocytes affects chondrocyte metabolism by interfering with the mitochondrial oxidation of energetic substrates that, in turn, are metabolically processed via the anaerobic glycolysis with consequent energy loss and increased production of radical oxygen species. Second, both conditions foster a relevant impairment of cellular metabolism and function, resulting in chondrocyte necrosis and apoptosis and activating synovial inflammation. Mitochondrial dysfunction, observed in experimental conditions of hyperglycemia, is also associated with a diminished synthesis of type 2 collagen coupled with enhanced proteolysis of cartilage proteoglycans by neutrophil-derived metalloprotease.

The results of a pilot cross-sectional study assessing the levels of synovial inflammation in patients with POA found that patients with an established diagnosis of type 2 diabetes, compared to euglycemic individuals, had more relevant signs of synovitis expressed as erythrocyte extravasation and a higher number of mast cells and macrophages. At the same time, the author found less microvascular representation in synovial tissues of patients with diabetes compared to those without and a positive relationship between the magnitude of synovial inflammation and the severity of knee pain (Eitner et al.).

Overall, these results indicate that hyperglycemia, impaired glucose control, and related dysmetabolic dysfunction have a pathophysiological role in maintaining and exacerbating synovial inflammation in POA. Strategies to improve glucose control are expected to reduce synovial inflammation and related outcomes.

The results of a Mendelian randomization analysis demonstrate a causal positive relationship between serum ferritin levels and the risk of POA in a European population, indicating a specific genetic background of iron dysmetabolism as a predisposing factor of the disease (Wang et al.). The results of this study may have a relevant fallout in specific fields of research and policy of prevention in primary care, as a simple laboratory exam can identify at-risk patients and sketch them out for proper pharmacological and non-pharmacological management.

A cross-sectional analysis from the National Health and Nutrition Examination Survey found that the body roundness index, a more sensitive anthropometric value than the body mass index in estimating the amount of visceral adipose tissue (17), positively correlated with established POA (18). The results of this study are substantially in line with current evidence suggesting that weight excess, particularly visceral adipose tissue excess, and background inflammation (well expressed by the body roundness index) are risk factors for POA.

Tian et al. provide an update on the efficacy and safety of mesenchymal stem cells, compared to placebo (saline), hyaluronic acid, glucocorticoids, platelet-rich plasma, or other non-surgical approaches, in the treatment of knee POA. The results of this systematic review with meta-analysis provided evidence that intra-articular administration of stem cells, administered at variable doses (single vs. double injections, from 1 to 100 million cells per injection), improved several clinical domains of knee OA such as pain, signs of inflammation, and joint function. Besides the promising results so far obtained, more investigation is needed to elucidate better the efficacy, effectiveness, and safety of regenerative therapy in POA. There is a real need for precise protocols of time and dose administration (dose-finding), as well as specific study designs to better analyze long-term outcomes in POA.

Halabitska et al. suggested a potential link between chronic pancreatitis and OA as a complication, based on findings from observational studies. Their analysis revealed that chronic pancreatitis exacerbates the progression of POA, as evidenced by assessments using the Western Ontario and McMaster Universities (osteo)Arthritis (WOMAC) index, body mass index, and C-reactive protein. Moreover, chronic pancreatitis affects the nutritional status negatively. However, due to the relatively small sample size of the study, these findings require validation through larger-scale observational research.

To conclude, the Research Topic elucidates the existence of specific groups of metabolic patients at high risk of developing POA, the potential mechanisms involved in such relationship, and some diagnostic tools to characterize at-risk patients for adequate management. It also highlights the need for more investigation in basic and clinical research, especially considering specific pharmacological interventions with modern agents for improving glucose control and reducing body weight.
